# Supplementary material for: Post COVID-19 vaccination side effects and associated factors among vaccinated health care providers in Oromia region, Ethiopia in 2021
Source: PLoS One. 2022 Dec 8;17(12):e0278334. doi: 10.1371/journal.pone.0278334 (PMC9731451; doi:10.1371/journal.pone.0278334)
Supplement: S1 Table — (DOCX) [file pone.0278334.s001.docx]

S1 Table: Socio-demographic characteristics of respondents with comorbidities for the study of post COVID-19 vaccine evaluations in Oromia region, Ethiopia, 2021.

| **Characteristics** | **Frequency** | **Percentage** |
| --- | --- | --- |
| **Sex** |  |  |
| Male | 462 | 50.7 |
| Female | 450 | 49.3 |
| **Age** |  |  |
| 20-29 | 325 | 35.6 |
| 30-39 | 439 | 48.1 |
| 40-49 | 118 | 12.9 |
| >=50 | 30 | 3.3 |
| **Marital status** |  |  |
| Single | 278 | 30.5 |
| Married | 581 | 63.7 |
| Separated | 18 | 2.0 |
| Divorced | 24 | 2.6 |
| Widowed | 11 | 1.2 |
| **Profession** |  |  |
| Physician(MD) | 112 | **12.3** |
| Pharmacist | 137 | **15.0** |
| Health officer | 41 | 4.5 |
| Nurse | 328 | 36.0 |
| Midwifery | 165 | 18.1 |
| Anesthetist | 33 | 3.6 |
| Laboratory | 96 | 10.5 |
| **Qualifications** |  |  |
| Diploma | 102 | 11.2 |
| Degree | 723 | 79.3 |
| Masters and above | 60 | 6.6 |
| Specialty and above | 27 | 3.0 |
| **Religion** |  |  |
| Orthodox | 474 | 52.0 |
| Muslim | 216 | 23.7 |
| Protestant | 195 | 21.4 |
| Catholic | 21 | 2.3 |
| Other | 6 | 0.7 |
| BMI in kg/m^2^ |  |  |
| <18.5 | 42 | 4.6 |
| 18.5-24.99 | 637 | 69.8 |
| >=25 | 233 | 25.5 |
