# Supplementary material for: Post COVID-19 vaccination side effects and associated factors among vaccinated health care providers in Oromia region, Ethiopia in 2021
Source: PLoS One. 2022 Dec 8;17(12):e0278334. doi: 10.1371/journal.pone.0278334 (PMC9731451; doi:10.1371/journal.pone.0278334)
Supplement: S2 Table — (DOCX) [file pone.0278334.s002.docx]

S2 Table: Chronic health problems of the respondents for the study of post COVID-19 vaccine evaluations in Oromia region, Ethiopia, 2021.

| **Characteristics** | **Frequency** | **Percentage** |
| --- | --- | --- |
| **Do you have any chronic health problems** |  |  |
| Yes | 57 | 6.3 |
| No | 855 | 93.8 |
| **Comorbidities** |  |  |
| Diabetes | 26 | 45.6 |
| Hypertension | 16 | 28.1 |
| Heart disease | 1 | 1.8 |
| Asthma | 13 | 22.8 |
| Chronic respiratory disease | 1 | 1.8 |
| Liver disease | 1 | 1.8 |
| **Currently taking treatment** |  |  |
| Yes | 42 | 73.7 |
| No | 15 | 26.3 |
