# Supplementary material for: Post COVID-19 vaccination side effects and associated factors among vaccinated health care providers in Oromia region, Ethiopia in 2021
Source: PLoS One. 2022 Dec 8;17(12):e0278334. doi: 10.1371/journal.pone.0278334 (PMC9731451; doi:10.1371/journal.pone.0278334)
Supplement: S3 Table — (DOCX) [file pone.0278334.s003.docx]

**S3 Table: Respondents COVID-19 status before the vaccination for the study of post COVID-19 vaccine evaluations in Oromia region, Ethiopia, 2021.**

| **Characteristics** | **Frequency** | **Percentage** |
| --- | --- | --- |
| **Ever tested for COVID-19** |  |  |
| Yes | 398 | 43.6 |
| No | 514 | 56.4 |
| **COVID-19 test result** |  |  |
| Positive | 136 | 34.2 |
| Negative | 262 | 65.8 |
| **Where did you get treatment** |  |  |
| Home based | 82 | 60.3 |
| COVID-19 treatment center | 38 | 27.9 |
| Isolation center | 16 | 11.8 |
| **Symptoms** |  |  |
| Severe Shortness of breath admission to ICU with oxygen | 26 | 19.1 |
| Severe shortness of breath without ICU admission with oxygen | 45 | 33.1 |
| Severe headache | 70 | 51.5 |
| Severe cough | 90 | 66.2 |
| Other | 8 | 5.9 |
| **Any family members who get infected with COVID-19** |  |  |
| No | 735 | 80.6 |
| Yes | 177 | 19.4 |
| **Any family loss because of COVID-19** |  |  |
| No | 892 | 97.8 |
| Yes | 20 | 2.2 |
