# Supplementary material for: Post COVID-19 vaccination side effects and associated factors among vaccinated health care providers in Oromia region, Ethiopia in 2021
Source: PLoS One. 2022 Dec 8;17(12):e0278334. doi: 10.1371/journal.pone.0278334 (PMC9731451; doi:10.1371/journal.pone.0278334)
Supplement: S4 Table — (DOCX) [file pone.0278334.s004.docx]

S4 Table: Respondents behavioral factors for the study of post COVID-19 vaccine evaluations in Oromia region, Ethiopia, 2021.

| **Characteristics** | **Frequency** | **Percentage** |
| --- | --- | --- |
| **smoke cigarettes** |  |  |
| Yes | 25 | 2.7 |
| No | 887 | 2.7 |
| **Drink alcoholic drinks** |  |  |
| Yes | 183 | 20.1 |
| No | 729 | 79.9 |
| **Frequency of alcohol drink** |  |  |
| Daily | 15 | 8.0 |
| 5-6 days per week | 14 | 7.5 |
| 1-4 days per week | 35 | 18.7 |
| 1-3 days per month | 68 | 36.4 |
| Less than once a month | 35 | 29.4 |
| **Chew Khat** |  |  |
| Yes | 80 | 8.8 |
| No | 832 | 91.2 |
| **Frequency of chew khat** |  |  |
| Daily | 22 | 27.5 |
| 5-6 days per week | 16 | 20.0 |
| 1-4 days per week | 12 | 15.0 |
| 1-3 days per month | 8 | 0.9 |
| Less than once a month | 22 | 27.5 |
| **Engage in vigorous intensity activity** |  |  |
| Yes | 62 | 7.3 |
| No | 845 | 92.7 |
| Number of days per week vigorous intensity activity |  |  |
| 1 | 5 | 7.5 |
| 2 | 27 | 40.3 |
| 3 | 19 | 28.4 |
| 4 | 13 | 19.4 |
| 5 | 3 | 4.5 |
| **Work involve moderate-intensity activity** |  |  |
| Yes | 124 | 13.6 |
| No | 788 | 86.4 |
| Number of days per week vigorous intensity activity |  |  |
| 2 | 22 | 17.7 |
| 3 | 17 | 13.7 |
| 4 | 13 | 10.5 |
| 5 | 63 | 50.8 |
| 6 | 6 | 4.8 |
| 7 | 3 | 2.4 |
