# Supplementary material for: Post COVID-19 vaccination side effects and associated factors among vaccinated health care providers in Oromia region, Ethiopia in 2021
Source: PLoS One. 2022 Dec 8;17(12):e0278334. doi: 10.1371/journal.pone.0278334 (PMC9731451; doi:10.1371/journal.pone.0278334)
Supplement: Attachment — Submitted filename: comment for authors.docx [file pone.0278334.s006.docx]

**Comments for authors**

**Abstract part:**

- “Preventive measures taken toward preventing COVID-19 infection were decreased post-COVID-19 vaccination” this statement is not clear. Make it clear and rewrite again.
- In the result part of abstract we expected you to describe factors with its statistical analysis and significance.

**Introduction part:**

- Why you select Oromia region for you study and health professionals as study participants?
- How have justified whether there was a gap and what it is?

# Methods:

- Under Study area and period; Better to mention health-related data of the region (number of health facilities with its different levels, health professionals, covid-19 centers and…etc)
- You didn’t describe or include your study population?
- Not clearly described inclusion criteria of your study.
- Under sample size and sampling procedures: Why did you take the p-value from Saudi Arabia, out of African content? You can use the p-value from African countries which you discussed in the discussion section. Mostly non-response rates should be 5-10%, why do you consider 15%? Need justification.
- How many health facilities were selected for study?
- From which disciplines (professions) did health care providers have participated in the study?
- How many health care providers are taken from each discipline or profession
- Please clearly describe the detailed sample size calculation and indicate how you took study participants.
- Need to describe sample size calculation formula and steps
- How did you consider the participants who took the covid-19 vaccine other than Asterzenica?

**Under data collection procedures you have to clearly describe the following concerns.**

- You have to cite all sources you used to develop your tool.
- How many parts do your tools have? No description of it.
- No description of about dependent and independent variables
- What is your major outcome or primary outcome?
- What type of items your tool has and how do you collect them from study participants?
- How many questions did you use to assess your objectives?
- From where you collected your data (document, interview, self-administered questioner, observations…..?
- How many data collectors have participated in the study?
- Does your study contain an observation part or checklist guided?
- Did data collectors participate in sampling processes?
- Who delivered the training for data collectors?
- On how many populations do you conduct your pretest?
- From where you draw the population for the pretest
- As you collected data by Likert scale it needs to do Cronbach's alpha to check your data's internal consistency. So, check and perform it.

**Results**

- It is good and presented in a good manner, but Socio-demographic characteristics, Chronic comorbidities conditions of participants, COVID-19 status of the participants before the vaccination, and behavioral factors you cited as S1, S2, S3, S4, what does it implies or why you didn’t put their result? Don’t copy all findings presented in the table or figures to the description part, only focus on the major result.
- Why being orthodox or other religion did significantly associate with post- covid 19 vaccination side effects? You can interpret this part? It is not likely to happen? Need to review your data again.

**Discussion and conclusion**

**Discussion**

- You are not expected to discuss all your findings, Focus only on the major findings.
- You didn’t discuss the factors responsible for **Post COVID-19** vaccination and its significance. You missed very important points and focused on minor findings.
- You didn’t address about statistically significance of factors responsible for Post COVID-19 vaccination
- You mentioned “The study tried to compare the events before and after the vaccine preventive measures toward COVID-19 infection” Is it you conducted a comparative study?

**Conclusion**

- You should describe only the major findings and factors you identified
- No need of describing all figures of your findings
- Be specific and conclusive.
- “In addition, further research on the cold chain of the vaccine, the reported side effect may be due to the problem related to the storage, transportation, and problem-related to the administration of the vaccine”. Did you investigate these factors?

Generally, this paper needs a detailed review and major changes. As it is written in an unorganized way, especially in methods, discussion, and conclusion parts.
