# Supplementary material for: Post COVID-19 vaccination side effects and associated factors among vaccinated health care providers in Oromia region, Ethiopia in 2021
Source: PLoS One. 2022 Dec 8;17(12):e0278334. doi: 10.1371/journal.pone.0278334 (PMC9731451; doi:10.1371/journal.pone.0278334)
Supplement: Attachment — Submitted filename: Response to Reviewrs.docx [file pone.0278334.s007.docx]

**Comments for authors**

**Abstract part:**

- “Preventive measures taken toward preventing COVID-19 infection were decreased post-COVID-19 vaccination” this statement is not clear. Make it clear and rewrite again.

Response: Rewritten as ‘‘ COVID-19 infection preventive protocols like keeping distance, hand wash using soap, wearing mask and using sanitizer were decreased post vaccination’’.

- In the result part of abstract we expected you to describe factors with its statistical analysis and significance.

Response: Comment accepted and included

**Introduction part:**

- Why you select Oromia region for you study and health professionals as study participants?
  - Response: Oromia region is selected due administrative issues and, the reason for selecting the health professional as study participant was because of the country gave priorities of providing vaccination to the health workforce and prioritized population groups. So it was difficult to include all the general population.
- How have justified whether there was a gap and what it is?
  - Response: I think the gap is mentioned in this section….ie. the following statements and others ,,, ‘‘Moreover, the rapid development of vaccines casts doubt on safety. Previously, the rapid developments of the vaccines have been linked to different adverse issues (11, 12). For example, the swine flu vaccine increased the risk of Guillain-Barre syndrome (13).’’

# Methods:

- Under Study area and period; Better to mention health-related data of the region (number of health facilities with its different levels, health professionals, covid-19 centers and…etc)

Response: Included as per the comment.

- You didn’t describe or include your study population?
- Not clearly described inclusion criteria of your study.

Response: I think it is clearly stated under source population

- Under sample size and sampling procedures: Why did you take the p-value from Saudi Arabia, out of African content? You can use the p-value from African countries which you discussed in the discussion section. Mostly non-response rates should be 5-10%, why do you consider 15%? Need justification.

Response: The concern is accepted. Since the disease was new, we were unable to get the published studies regarding the disease during proposal development time at in Ethiopia as well in Africa. So used the Saudi Arabia study since no statistical prohibition of using abroad study result. Regarding the response rate of using 15%, by assuming that non-response will be high due to the nature of the study. Fortunately we were lucky the response rate was 97.1%

- How many health facilities were selected for study?

Response: Six Health facilities (Asella Teaching and Referral Hospital=260 participants, Bokoji Hospital=50 participants, Adama Medical Hospital College=250 participants, Mojo Hospital=50 participants, Bishoftu Hospital =100 participants, and Shashemene Referral Hospital==202 participants

- From which disciplines (professions) did health care providers have participated in the study?

Response: All professionals= check supplementary material 1(S1)

- How many health care providers are taken from each discipline or profession

Response: Check supplementary material 1(S1)

- Please clearly describe the detailed sample size calculation and indicate how you took study participants.
  - Response: I think this section is clearly stated.
- Need to describe sample size calculation formula and steps
  - Response: Sample size is calculated using Epi Info software; I think no need to put the formula.
- How did you consider the participants who took the covid-19 vaccine other than Asterzenica?
  - Response: They were included but the numbers are insignificant

**Under data collection procedures you have to clearly describe the following concerns.**

- You have to cite all sources you used to develop your tool.
  - Response: Cited in the revised manuscript
- How many parts do your tools have? No description of it.
  - Response: The tools have eight (8) parts.
- No description of about dependent and independent variables
  - Response: The comment accepted
- What is your major outcome or primary outcome?
  - Response: The major outcome is the reported side effect
- What type of items your tool has and how do you collect them from study participants?
  - Response: The response is given under the comment, ‘‘How many parts do your tools have?’’
- How many questions did you use to assess your objectives?
  - Response: Two questions (side effets in the 1^st^ dose or 2^nd^ dose as Yes /No)
- From where you collected your data (document, interview, self-administered questioner, observations…..?
  - Response: The data were collected using interview administered
- How many data collectors have participated in the study?
  - Response: Twenty one (21) data collectors.
- Does your study contain an observation part or checklist guided?
  - Response: It did not include observation checklist, it is mentioned as limitation of the study.
- Did data collectors participate in sampling processes?
  - Response: No! the supervisors participated
- Who delivered the training for data collectors?
  - Response: The investigators
- On how many populations do you conduct your pretest?
- From where you draw the population for the pretest
  - Response: The pretest was conducted on 48 individuals (5% of the total samples) selected from Asella Health Center(13 subjects), Eteya Health Center(1 0 subjects), Sagure Health Center(10 and Adama Health Center(15 subjects)
- As you collected data by Likert scale it needs to do Cronbach's alpha to check your data's internal consistency. So, check and perform it.

Response: Thanks for your suggestion but only one question is collected using likert scale, so need to check chronbach’s alpha. The question was’

‘Do you agree that taking the vaccine will help to prevent the pandemic?’ (Strongly agree, Agree, Neutral, Disagree, Strongly disagree).

**Results**

It is good and presented in a good manner, but Socio-demographic characteristics, Chronic comorbidities conditions of participants, COVID-19 status of the participants before the vaccination, and behavioral factors you cited as S1, S2, S3, S4, what does it implies or why you didn’t put their result?

Response: S1, 2, 3 … Is to mean supporting information. It is cited to decrease the number of tables cited to respect the submission guideline. Their result already presented in the manuscript.

Don’t copy all findings presented in the table or figures to the description part, only focus on the major result.

Response: comment accepted

- Why being orthodox or other religion did significantly associate with post- covid 19 vaccination side effects? You can interpret this part? It is not likely to happen? Need to review your data again.
  - Response: Religion could be a factor

**Discussion and conclusion**

**Discussion**

- You are not expected to discuss all your findings, Focus only on the major findings.
- You didn’t discuss the factors responsible for **Post COVID-19** vaccination and its significance. You missed very important points and focused on minor findings.
- You didn’t address about statistically significance of factors responsible for Post COVID-19 vaccination
  - Response: Dear reviewer thank you for your concern but the factors already discusses
- You mentioned “The study tried to compare the events before and after the vaccine preventive measures toward COVID-19 infection” Is it you conducted a comparative study?
  - Response: It is not to indicate comparative study. If you see the majority of the study showed results of 1^st^ dose

**Conclusion**

- You should describe only the major findings and factors you identified
- No need of describing all figures of your findings
- Be specific and conclusive.
- “In addition, further research on the cold chain of the vaccine, the reported side effect may be due to the problem related to the storage, transportation, and problem-related to the administration of the vaccine”. Did you investigate these factors?
  - Response: The cold chain not investigated but some of the reported side effect may be due to improper handling of the vaccine. This is our suggestion. It could be a factor. The rest comment accepted for change.
